# Periodontal disease prevalence, extent, and risk associations in untreated individuals

**DOI:** 10.1002/cre2.526

**Published:** 2022-01-10

**Authors:** Yasmine N. Alawaji, Abdulsalam Alshammari, Nesrine Mostafa, Ricardo M. Carvalho, Jolanta Aleksejuniene

**Affiliations:** ^1^ Department of Preventive Dental Science, College of Dentistry King Saud bin Abdul‐Aziz University for Health Sciences Riyadh Saudi Arabia; ^2^ King Abdullah International Medical Research Center National Guard Health Affairs Riyadh Saudi Arabia; ^3^ Department of Oral Health Sciences, Faculty of Dentistry The University of British Columbia Vancouver British Columbia Canada; ^4^ Department of Oral Biological and Medical Sciences, Faculty of Dentistry The University of British Columbia Vancouver British Columbia Canada

**Keywords:** natural history, periodontal diseases, prevalence, risk factors

## Abstract

**Objectives:**

to examine the prevalence, extent, and risk associations of untreated periodontitis.

**Materials and Methods:**

A purposive sample of subjects who were never treated for periodontal conditions was clinically examined after collecting information about their sociodemographic characteristics, medical conditions, oral health behaviors, perceived stress, and perceived social support.

**Results:**

A total of 431 subjects were recruited (response rate, 97.0%), and their mean age (SD) was 35.4 (13.3) years. Overall, high plaque levels were observed in all untreated individuals. The prevalence of periodontitis and severe (stage III/IV) periodontitis using the American Academy of Periodontology and European Federation of Periodontology (AAP/EFP) classification were 85.4% and 48.5%, respectively. The prevalence of moderate‐severe and severe periodontitis using the definitions of the Centers for Disease Control and Prevention (CDC) and AAP were 78.4% and 31.1%, respectively. The extent of periodontitis expressed as mean% of clinical attachment loss (CAL) ≥ 3 mm and CAL ≥ 5 mm were 34.9% and 14.4%, respectively, while the mean% of a periodontal probing depth (PPD) ≥4 mm and PPD ≥6 mm were 22.0% and 9.2%, respectively. Risk determinants associated with AAP/EFP periodontitis after the adjustment for other variables were age ≥35 years (odds ratio [OR] = 11.5) and lower income (OR = 2.5). Adjusted risk associations with stage II/IV periodontitis included age ≥35 years (OR = 8.2), males (OR = 2.5), lower income (OR = 2.3), and lower perceived stress (OR = 2.0). Adjusted risk associations with CDC/AAP moderate‐severe periodontitis included age ≥35 years (OR = 12.0), lower income (OR = 2.1), and current cigarette smoking (OR = 4.2). Adjusted risk associations with CDC/AAP severe periodontitis included age ≥35 years (OR = 4.5), males (OR = 1.9), lower education (OR = 2.0), lower income (OR = 1.7), uncontrolled diabetes mellitus (OR = 2.0), and current cigarette smoking (OR = 2.3).

**Conclusions:**

The prevalence and extent of periodontitis were high in untreated subjects. Risk associations with untreated periodontitis included age ≥35 years, males, lower income, lower education, current cigarette smoking, uncontrolled diabetes mellitus, and lower perceived stress.

## 1 INTRODUCTION

In epidemiology, the natural history of a disease is defined as the “uninterrupted progression of disease in an individual from the moment that the disease was initiated by exposure to a causal agent” (Bhopal, 2016; Jewell, 2016). The exposures (causes/risks) can result in any of the following responses: a disease outcome is not evident, the outcome is damaging but repairable, outcome manifests as an illness that the body's immunity can contain, or the exposure leads to long‐term damage or even mortality. Studying the natural course of the disease without an external intervention and its associated exposures/risks is important for planning disease prevention or treatment measures.

Earlier studies focused on periodontal disease in untreated populations by recruiting individuals who visited dental offices (Becker et al., 1979; Goodson et al., 1982; Lindhe et al., 1983) or targeting untreated populations who resided in rural areas; thus, they did not have access to regular dental care or preventive dental programs (Dowsett et al., 2001; Dowsett, Eckert, et al., 2002; Dowsett, Kowolik, et al., 2002; Loe et al., 1978; Löe et al., 1986; Timmerman et al., 1998, 2000; Van der Velden et al., 2006). These earlier studies focused on examining onset and progression rate of untreated periodontitis by conducting longitudinal assessments in the absence of plaque control or periodontal treatment. Despite high plaque and calculus levels among the untreated subjects, the periodontal disease severity and rate of progression varied among the untreated individuals, indicating different susceptibility levels to periodontitis. Only two previous studies evaluated the risk associations with untreated periodontitis in tea workers (Neely et al., 2001; Van der Velden et al., 2006). In these two studies, medical conditions, socioeconomic status, and psychological determinants were not examined for their potential associations with periodontitis. Thus, our current knowledge of the risk associations of untreated periodontitis remains limited.

Studies of periodontal diseases in the literature have restricted their inclusion to younger (Albandar et al., 1997; Goldberg et al., 1985) or older age groups (Albandar et al., 1999; Baelum et al., 1988; P. I. Eke et al., 2018). Such age‐related restrictions can preclude direct comparisons among different age cohorts. This restriction was traditionally supported by different periodontal disease diagnoses based on age (Albandar et al., 1997; American Academy of Periodontology, 1989, 2015; Caton et al., 2018; Highfield, 2009). However, the current classification system of the American Academy of Periodontology and the European Federation of Periodontology (AAP/EFP) designates patients with periodontitis under one main entity (Caton et al., 2018; Papapanou et al., 2018). Then, a matrix of staging, grading, and descriptors, which considers age, is used to reach the clinical periodontal diagnosis (Tonetti et al., 2018).

The aims of the current study were to: (1) examine the prevalence and extent of the risk associations of untreated periodontitis; (2) compare the periodontal findings of younger and older individuals; and (3) test the application of the AAP/EFP new clinical classification system in epidemiological studies.

## 2 MATERIALS AND METHODS

Ethics approval was obtained from the King Abdullah International Medical Research Center (H‐01‐R‐005). Data were collected from September to December 2019. A purposive sample of untreated subjects was recruited consecutively from the screening dental clinics at the College of Dentistry, King Saud bin Abdul‐Aziz University for Health Sciences in Riyadh, Saudi Arabia. This public dental school was established less than 10 years ago and is located at the eastern boundary of Riyadh city. Patients visiting this dental school mainly reside in rural areas surrounding the city. The subjects who agreed to participate in the study signed a consent form, and all included subjects were able to communicate in Arabic or English.

Inclusion criteria: Dentate or partially dentate individuals who had a previous dental care history limited to emergency dental treatment and were never treated for periodontal conditions in their lifetime (never received non‐surgical or surgical periodontal treatment). Exclusion criteria: Patients who received any past periodontal treatment (non‐surgical or surgical), had mental illness/conditions that prevented them from participating, had unstable medical conditions, needed prophylactic antibiotics or other medications before clinical examinations; fully edentulous subjects; and individuals with acute dental conditions that required urgent care such as an abscess, cellulitis, or diseases affecting the jawbones including cysts and neoplasms.

### 2.1 Questionnaires

The study questionnaires were translated into Arabic using the forward–backward method. Illiterate subjects were assisted in completing the questionnaires. The questionnaire inquired about age, sex, ethnicity, formal education level, monthly household income measured in the local currency (Saudi Riyal), medical conditions, use of medications, daily toothbrushing habits, use of interdental cleaning aids, reason for visiting the dental school clinic, frequency of dental visits, past dental history, smoking status, perceived stress using the 10‐item Perceived Stress Scale (PSS) (Cohen et al., 1983, 1994), and perceived social support using the 12‐item Multidimensional Scale of Perceived Social Support (MSPSS) (Zimet et al., 1988). All medical conditions were self‐reported. Subjects who identified as diabetic were asked about their glycemic control and self‐reported their most recent glycated hemoglobin (HbA1c) level. Perceived stress and perceived social support measures used a visual analog scale ranging from 0% to 100%. A self‐assessment of oral hygiene and periodontal health was performed using a 5‐point Likert scale ranging from very poor to excellent.

### 2.2 Clinical examinations

A certified periodontist conducted all of the clinical assessments, which consisted of a full‐mouth examination of 28 teeth, excluding the third molars. Manual probing was performed at six sites per tooth, and all measurements were rounded to the nearest millimeter. The periodontal examination included the following: full‐mouth plaque scores (FMPS) based on the presence of plaque at the site level, periodontal probing depth (PPD), recession (REC), clinical attachment loss (CAL), and bleeding on probing (BOP).

### 2.3 Periodontitis outcomes

For periodontitis prevalence, the AAP/EFP diagnostic criteria were used (23,24) in which gingivitis was defined as BOP at ≥10% of the sites (Trombelli et al., 2018). AAP/EFP periodontitis was defined using staging and grading in which staging was mainly assigned based on the most severe CAL at ≥2 interproximal sites or midbuccal sites with a CAL ≥3 mm and PPD ≥3 mm at ≥2 teeth (Tonetti et al., 2018). Stage I was based on CAL of 1–2 mm; stage II was a CAL range of 3–4 mm; and stage III/IV was a CAL of ≥5 mm. Complexity factors that are considered stage modifiers include the PPD, tooth loss, or presence of <20 remaining teeth. Grading was assigned based on modification of the risk by glycemic control of diabetes mellitus and cigarette smoking status. Additionally, periodontitis case definitions for epidemiological studies by the Centers for Disease Control and Prevention (CDC) and AAP were used (P. Eke et al., 2012; Page & Eke, 2007). CDC/AAP moderate to severe periodontitis was defined as a CAL ≥4 mm at ≥2 interproximal sites (not at the same tooth) or a PPD ≥5 mm at ≥2 interproximal sites (not at the same tooth). CDC/AAP severe periodontitis was defined as a CAL ≥6 mm at ≥2 interproximal sites (not at the same tooth) and a PPD ≥5 mm at ≥1 interproximal site (can be at one of the two teeth with CAL). The extent of periodontitis was defined as the mean percentage of CAL or PPD at different thresholds (Holtfreter et al., 2015).

To study the risk associations with periodontitis, we selected four outcomes: (1) AAP/EFP total periodontitis (stage I/II/III/IV); (2) AAP/EFP severe periodontitis (stage III/IV); (3) CDC/AAP moderate to severe periodontitis; and (4) CDC/AAP severe periodontitis.

### 2.4 Sample size

The minimum sample size was calculated based on the primary data of the first 100 subjects using the G*power program version 3.1.9.2 (Faul et al., 2009). To calculate the minimum sample size for multivariate logistic models, we selected a power of 0.8 and an *α* level of .05. For CDC/AAP moderate‐severe periodontitis, the estimated minimum sample size was 350 based on the current smoking odds ratio (odds ratio [OR] = 2.9), current smoking frequency of 22.0%, and *R^2^* = 0.450. The estimated sample for CDC/AAP severe periodontitis was 330 based on current smoking (OR = 2.6), frequency of 22.0%, and *R* ^2^ = 0.263. To compensate for the possibility of missing data and ensure an adequate number/frequency of cases per determinant, we recruited an additional 81 subjects.

### 2.5 Statistical analyses

All statistical analyses were performed at the subject level using SPSS version 27.0 software (IBM, Chicago, IL, USA). The reliability of the subjects' responses was tested using Cohen's Kappa test, which compares repeated responses to an education‐related question placed in two different places within the same questionnaire. The internal consistency of the subjects' responses was tested using Cronbach's alpha for the 10‐item PSS and the 12‐item MSPSS. To assess intra‐examiner reliability, duplicate clinical measurements were performed in 10 randomly selected subjects. Intra‐examiner agreement was calculated using the intraclass correlation coefficient (ICC) to compare a duplicate recording of the total number of sites at the two CAL thresholds of ≥3 mm and ≥5 mm.

The distribution of the sample background characteristics was described using univariate analysis. Age in years was measured as a continuous variable and divided into younger and older groups based on the sample's mean age of 35 years. The distribution of periodontal disease prevalence and extent was stratified by age to compare the periodontal findings between groups.

For the risk associations with periodontitis, we used the four abovementioned periodontitis outcomes as dependent variables. Bivariate logistic regression was used to calculate the unadjusted risk associations, while multivariate logistic regression analyses were used to estimate the adjusted risk associations of periodontitis. The following independent variables were included in the logistic models: (1) age: <35 years versus ≥35 years; (2) sex: male versus female; (3) level of formal education: less than high school compared with more than high school; (4) household monthly income: lower income (<5000 Saudi Riyal) compared with higher income (≥5000 Saudi Riyal); and (5) self‐reported diabetes mellitus without glycemic control compared with the rest of the sample since only two subjects reported glycemic control; (6) self‐reported obesity compared with non‐obesity; (7) cigarette smoking: dummy variables were created and current and former smokers were compared with never‐smokers; (8) perceived stress; and (9) perceived social support were initially measured as continuous variables and then divided into lower or higher categories based on the sample's mean scores.

We excluded from logistic regression analyses: (1) any variable that lacked variation; or (2) had an insufficient number of subjects, that is, less than 10 subjects per variable category. Multicollinearity diagnostics were examined in all multivariate regression models to validate the findings.

## 3 RESULTS

Of the 515 new patients approached at screening dental clinics, 67 did not meet the inclusion criteria and 17 refused to participate (recruitment rate: 97%). Of the 431 recruited subjects, 185 (42.5%) were males and 246 (57.5%) were females. The age range was 13–80 years, with a mean (SD) age of 35.4 (13.3) years. Duplicate responses to the same question showed a high level of reliability as indicated by Cohen's Kappa of 0.99. For the 10‐item PSS and the 12‐item MSPSS, the internal consistency using Cronbach's alpha was 0.77 and 0.99, respectively. The intra‐examiner reliability of duplicate measurements using ICC was 0.99 for the total number of sites with a CAL ≥3 mm (95% confidence interval [CI], 0.94–0.99) and the ICC for the total number of sites with a CAL ≥5 mm was 0.97 (95% CI, 0.89–0.99).

The distribution of subjects' background data, oral health status, and oral behaviors is depicted in Tables 1 and 2. There was a lack of variation concerning the following determinants: ethnicity, where Arabs represented 90% of the sample; FMPS ≥25% was 96.3%, and the AAP/EFP gingivitis prevalence was 89.1%.

**Table 1:** Distribution of subjects' background characteristics

Determinants	*N* (%)
Age	
<35 years	215 (49.9)
35+ years	216 (50.1)
Ethnicity	
Arabic	388 (90.0)
Other	41 (10.0)
Education	
>High school	162 (37.6)
≤High school	266 (61.7)
Household monthly income	
Lower <5000 Saudi Riyal	239 (55.5)
Higher ≥5000 Saudi Riyal	189 (43.9)
Self‐reported medical conditions	
Diabetes mellitus, without glycemic control	63 (14.5)
Hypothyroidism	26 (6.0)
Obesity	80 (18.6)
Osteoporosis	27 (6.3)
Sex	
Males	183 (42.5)
Females	246 (57.1)
Smoking	
Never smoked	309 (71.7)
Former smoker	42 (9.7)
<10 cigarettes daily	30 (7.0)
≥10 cigarettes daily	48 (11.1)
Household members	
1–6	200 (46.3)
>6	228 (52.9)
Perceived stress	
Lower (≤42 score)	212 (49.2)
Higher (>42 score)	213 (49.4)
Perceived social support	
Lower (<69.5 score)	213 (49.4)
Higher (≥69.5 score)	212 (49.2)

**Table 2:** Subjects' oral behaviors and oral health status

Daily brushing, *N* (%)	272 (63.1)
Daily use of interdental aids, *N* (%)	137 (31.8)
Total number of remaining teeth, mean (SD)	23.8 (5.2)
Full mouth plaque scores ≥25%, *N* (%)	415 (96.3)
AAP/EFP gingivitis, *N* (%)	384 (89.1)
Self‐reported reasons for infrequent dental visits, *N* (%)	
Lack of time	233 (54.1)
Unavailable appointments at the public dental centers in their residential areas	191 (44.3)
Financial reasons	183 (42.5)
Fear from dental treatments	144 (33.4)
Lack of transportation	140 (32.5)
Lack of trust in dentists	73 (16.9)
Other reasons	57 (13.2)

Abbreviations: AAP, American Academy of Periodontology; EFP, European Federation of Periodontology.

**Figure 1:**
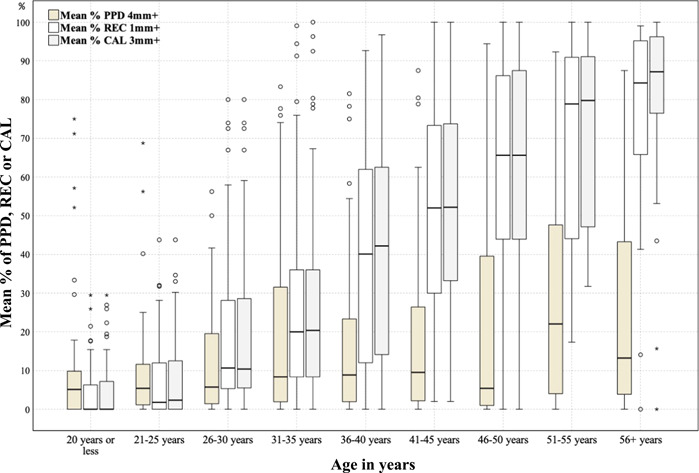
Distribution of periodontal disease extent by age cohort. Periodontal disease extent defined as mean % of periodontal probing depth (PPD), recession (REC), or clinical attachment loss (CAL)

**Figure 2:**
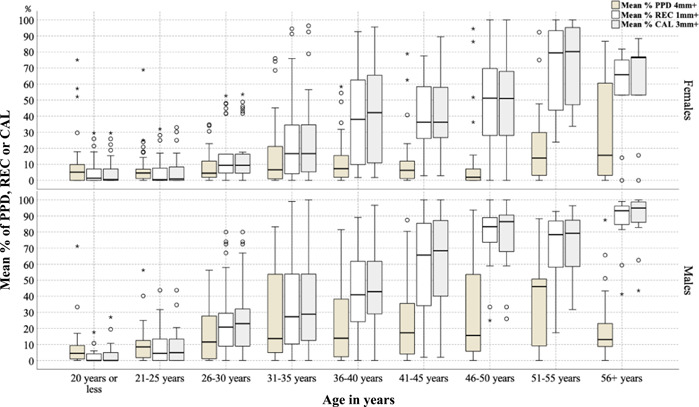
Periodontal disease extent by age cohort and patient sex

The distribution of periodontitis prevalence stratified by age is shown in Table 3. The prevalence of AAP/EFP total periodontitis and stage III/IV periodontitis were 85.4% and 48.5%, respectively. The prevalence of CDC/AAP moderate‐severe and severe periodontitis were 78.4% and 31.1%, respectively. The extent of periodontitis for CAL and PPD stratified by age is shown in Table 4. The mean % (SD) of sites with a CAL ≥3 mm was 34.9% (33.6), while the mean % (SD) of sites with a PPD ≥4 mm was 16.6% (22.0). The extent of periodontitis for different periodontal parameters, namely PPD, REC, and CAL, was compared among different age cohorts (Figure 1), and the REC and CAL increased with age, while the PPD did not have a clear pattern corresponding to an increase in age. The extent of PPD, REC, and CAL for different age cohorts are depicted by patient sex in Figure 2; males had a higher extent of PPD, REC, and CAL than females in most of the age cohorts.

**Table 3:** Distribution of periodontitis prevalence using the case definition criteria by the AAP/EFP and CDC/AAP

	Grade A	Grade B	Grade C	Total
AAP/EFP periodontitis prevalence, *N* (%)				
Stage I (mild)	34 (7.9)	3 (0.7)	3 (0.7)	40 (9.3)
Stage II (moderate)	89 (20.7)	7 (1.6)	23 (5.3)	119 (27.6)
Stage III (severe)	81 (18.8)	36 (8.4)	42 (9.8)	159 (36.9)
Stage IV (very severe)	24 (5.6)	9 (2.1)	17 (3.9)	50 (11.6)
	**<35 years** (*N*: 215)	**≥35 years** (*N*: 216)	**Total** (*N*: 431)	
Total periodontitis cases	159 (74.0)	209 (96.8)	368 (85.4)	
Total severe (stage III/IV) cases	49 (22.8)	160 (74.1)	209 (48.5)	
	**<35 years** (*N*: 215)	**≥35 years** (*N*: 216)	**Total** (*N*: 431)	
CDC/AAP periodontitis prevalence, *N* (%)				
Moderate‐severe periodontitis	132 (39.1)	206 (95.4)	338 (78.4)	
Severe periodontitis	28 (13.0)	107 (49.5)	135 (31.1)	

*Note*: Staging was mainly categorized based on the severity of CAL, where Stage I: CAL 1–2 mm, Stage II: CAL 3–4 mm, Stage III/IV: CAL 5 mm. The staging was then modified by the complexity factors such as periodontal probing depth, tooth loss and total of remaining teeth. Grading was applied in this study based on the modification of periodontitis risk by the glycemic control of subjects with diabetes mellitus and/or by smoking status. Grade A: non‐smokers, nondiabetic subjects, Grade B: smokers of <10 cigarettes daily and/or diabetic with glycemic control (HBA1c < 7%), Grade C: smokers of ≥10 cigarettes daily and/or diabetic subjects without glycemic control (HbA1c ≥ 7%).

Abbreviations: AAP, American Academy of Periodontology; CAL, clinical attachment loss; CDC, Centers for Disease Control and Prevention; EFP, European Federation of Periodontology.

**Table 4:** Periodontitis extent stratified by age for clinical attachment loss (CAL) and periodontal probing depth (PPD)

Periodontitis extent	<35 years (*N*: 215)	≥35 years (*N*: 216)	All subjects (*N*: 431)
CAL			
Mean (SD)% of CAL ≥3 mm	12.9 (19.1)	56.9 (30.4)	34.9 (33.6)
Mean (SD)% of CAL ≥4 mm	8.0 (15.8)	42.0 (32.7)	25.0 (30.8)
Mean (SD)% of CAL ≥5 mm	3.4 (11.3)	25.3 (30.9)	14.4 (25.7)
Mean (SD)% of CAL ≥6 mm	1.6 (7.8)	16.5 (26.2)	9.1 (20.7)
PPD			
Mean (SD)% of PPD ≥4 mm	12.0 (17.2)	21.1 (25.2)	16.6 (22.0)
Mean (SD)% of PPD ≥5 mm	2.2 (5.3)	9.4 (17.3)	5.8 (13.2)
Mean (SD)% of PPD ≥6 mm	0.6 (2.4)	5.2 (12.3)	2.9 (9.2)
Mean (SD)% of PPD ≥7 mm	0.3 (1.4)	2.9 (8.1)	1.6 (6.0)

**Figure 3:**
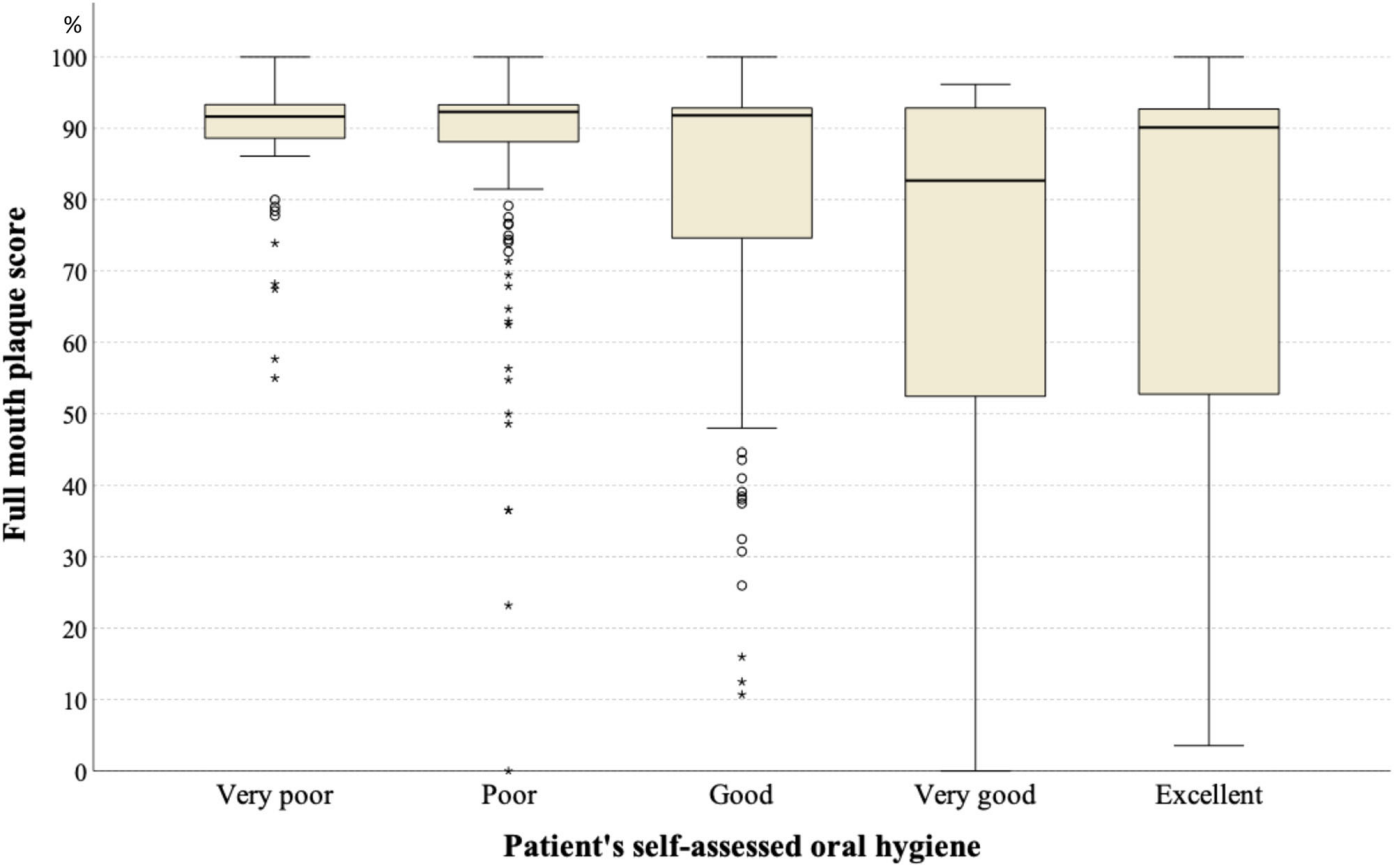
Self‐assessed oral hygiene versus assessed full‐mouth plaque scores

**Figure 4:**
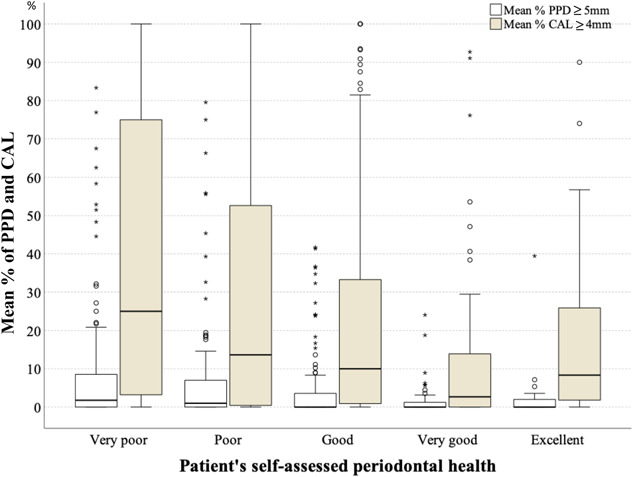
Self‐assessed periodontal health versus extent of periodontal disease defined as mean % of clinical attachment loss (CAL) ≥ 4 mm and mean % of periodontal probing depth (PPD) ≥ 5 mm

Self‐assessed oral hygiene by study subjects was compared with the clinically examined FMPS (Figure 3), which shows that most subjects who rated their oral hygiene as good to excellent had a high FMPS. The self‐assessed periodontal health was compared with the extent of patients with a CAL ≥4 mm and PPD ≥4 mm (Figure 4); some of the subjects who rated their periodontal health as good to excellent had high proportions of periodontitis.

For AAP/EFP total periodontitis cases (Table 5), the unadjusted, significantly associated risk determinants included age ≥35 years (OR = 10.5), male sex (OR = 2.2), lower income (OR = 1.8), and current cigarette smoking (OR = 3.7), while the adjusted risk associations included age ≥35 years (OR = 11.5) and lower income (OR = 2.5).

**Table 5:** Unadjusted and adjusted risk associations with total periodontitis defined using the AAP/EFP criteria

	AAP/EFP total periodontitis cases, **adjusted model summary: Nagelkerke *R* ^2^ = 0.252 (*p* < .001)**			
	Unadjusted odds ratio		Adjusted odds ratio	
Risk determinants	OR (95% CI)	*p* value	OR (95% CI)	*p* value
**Age (years)**				
<35 years^a^	1.0		1.0	
35+ years	10.5 (4.7, 23.7)	<.001	11.5 (4.5, 29.0)	<.001
**Sex**				
Females^a^	1.0		1.0	
Males	2.2 (1.2, 4.0)	.010	1.6 (0.7, 2.4)	.256
**Education**				
>High school^a^	1.0		1.0	
≤High school	1.0 (0.6, 1.8)	.880	0.6 (0.3, 1.2)	.126
**Household monthly income**				
Higher ≥5000 Saudi Riyal^a^	1.0		1.0	
Lower <5000 Saudi Riyal	1.8 (1.0, 3.2)	.043	2.5 (1.3, 4.9)	.007
**Diabetes mellitus**				
Other^a^	1.0		1.0	
No glycemic control	2.8 (0.9, 7.9)	.058	0.8 (0.2, 2.7)	.710
**Obesity**				
Non‐obese^a^	1.0		1.0	
Obese	1.6 (0.8, 3.6)	.213	1.0 (0.4, 2.6)	.949
**Cigarette smoking**				
Never‐smokers^a^	1.0		1.0	
Former smokers	1.3 (0.5, 3.4)	.622	0.7 (0.2, 0.2.1)	.470
Current smokers	3.7 (1.3, 10.4)	.015	2.5 (0.8, 8.6)	.134
**Perceived stress**				
Higher (≥42.1)^a^	1.0		1.0	
Lower (≤42)	1.4 (0.8, 2.4)	.222	1.2 (0.6, 2.3)	.565
**Perceived social support**				
Higher (≥69.5)^a^	1.0		1.0	
Lower (<69.5)	0.8 (0.5, 1.4)	.502	0.8 (0.4, 1.4)	.365

*Note*: Unadjusted odds ratio: one determinant (bivariate logistic regression), adjusted odds ratio: multiple determinants (multivariate logistic regression).

Abbreviations: AAP, American Academy of Periodontology; CI, confidence interval; EFP, European Federation of Periodontology; OR, odds ratio.

^a^ Reference category.

For AAP/EFP stage III/IV periodontitis (Table 6), the unadjusted significantly associated risk determinants included ≥35 years of age (OR = 9.7), male sex (OR = 2.7), lower education (OR = 2.2), lower income (OR = 1.7), uncontrolled diabetes mellitus (OR = 5.0), former smoking (OR = 3.3), current smoking (OR = 2.3), and lower perceived stress (OR = 1.6). The adjusted significantly associated determinants of stage III/IV periodontitis were age ≥35 years (OR = 8.2), male sex (OR = 2.5), lower income (OR = 2.3), and lower perceived stress (OR = 2.0).

**Table 6:** Unadjusted and adjusted risk determinants of stageIII/IV periodontitis using the AAP/EFP criteria

	AAP/EFP severe periodontitis (stage III/IV), adjusted model summary: Nagelkerke *R* ^2^ = 0.459 (*p* < .001)			
	Unadjusted odds ratio		Adjusted odds ratio	
Risk determinants	OR (95% CI)	*p* value	OR (95% CI)	*p* value
**Age (years)**				
<35 years^a^	1.0		1.0	
35+ years	9.7 (6.2, 15.0)	<.001	8.2 (5.0, 13.6)	<.001
**Sex**				
Females^a^	1.0		1.0	
Males	2.7 (1.8, 4.1)	<.001	2.5 (1.4, 4.6)	.003
**Education**				
>High school^a^	1.0		1.0	
≤High school	2.2 (1.5, 3.3)	<.001	1.6 (0.9, 2.8)	.063
**Household monthly income**				
Higher ≥5000 Saudi Riyal^a^	1.0		1.0	
Lower <5000 Saudi Riyal	1.7 (1.2, 2.5)	.006	2.3 (1.3, 3.9)	.002
**Diabetes mellitus**				
Other^a^	1.0		1.0	
No glycemic control	5.0 (2.6, 9.6)	<.001	1.8 (0.8. 3.8)	.154
**Obesity**				
Non‐obese^a^	1.0		1.0	
Obese	1.8 (1.1, 2.9	.023	1.3 (0.7, 2.5)	.357
**Cigarette smoking**				
Never‐smokers^a^	1.0		1.0	
Former smokers	3.3 (1.6, 6.8)	<.001	2.1 (0.8, 5.2)	.133
Current smokers	2.3 (1.4, 3.9)	.001	2.0 (0.9. 4.1)	.073
**Perceived stress**				
Higher (≥42.1)^a^	1.0		1.0	
Lower (≤42)	1.6 (1.1, 2.3)	.018	2.0 (1.2, 3.4)	.008
**Perceived social support**				
Higher (≥69.5)^a^	1.0		1.0	
Lower (<69.5)	1.3 (0.9, 2.0)	.132	1.7 (0.9, 2.7)	.051

*Note*: Unadjusted odds ratio: one determinant (bivariate logistic regression), adjusted odds ratio: multiple determinants (multivariate logistic regression).

Abbreviations: AAP, American Academy of Periodontology; CI, confidence interval; EFP, European Federation of Periodontology; OR, odds ratio.

^a^ Reference category.

For CDC/AAP moderate‐severe periodontitis (Table 7), the unadjusted and significantly associated risk determinants included age ≥35 years (OR = 13.0), male sex (OR = 2.0), lower education (OR = 1.7), uncontrolled diabetes mellitus (OR = 3.7), obesity (OR = 2.5), and current cigarette smoking (OR = 4.0); the adjusted significant risk determinants included age ≥35 years (OR = 12.0), lower income (OR = 2.1), and current cigarette smoking (OR = 4.2).

**Table 7:** Unadjusted and adjusted risk associations for the moderate‐severe periodontitis defined using the CDC/AAP criteria

	CDC/AAP moderate‐severe periodontitis, adjusted model summary: Nagelkerke *R* ^2^ = 0.327; (*p* < .001)			
	Unadjusted odds ratios		Adjusted odds ratios	
Risk determinants	OR (95% CI)	*p* values	OR (95% CI)	*p* values
**Age**				
<35 years^a^	1.0		1.0	
35+ years	13.0 (6.5, 25.9)	<.001	12.0 (5.6, 25.8)	<.001
**Sex**				
Females^a^	1.0		1.0	
Males	2.0 (1.2, 3.3)	.006	1.1 (0.6, 2.1)	.822
**Education**				
>High school^a^	1.0		1.0	
≤High school	1.7 (1.0, 2.7)	.003	0.9 (0.5, 1.6)	.786
**Household monthly income**				
Higher ≥5000 Saudi Riyal^a^	1.0		1.0	
Lower <5000 Saudi Riyal	1.5 (0.9, 2.4)	.101	2.1 (1.2, 3.7)	.014
**Diabetes mellitus**				
Other^a^	1.0		1.0	
No glycemic control	3.7 (1.4, 9.4)	.007	0.9 (0.3, 2.7)	.839
**Obesity**				
Non‐obese^a^	1.0		1.0	
Obese	2.5 (1.2, 5.2)	.015	1.6 (0.7, 3.7)	.300
**Cigarette smoking**				
Never‐smokers^a^	1.0		1.0	
Former smokers	2.2 (0.8, 5.7)	.113	1.5 (0.7, 3.7)	.466
Current smokers	4.0 (1.7, 9.4)	.002	4.2 (1.5, 11.7)	.006
**Perceived stress**				
Higher (≥42.1)^a^	1.0		1.0	
Lower (≤42)	1.4 (0.9, 2.2	.166	1.3 (0.8, 2.3)	.343
**Perceived social support**				
Higher (≥69.5)^a^	1.0		1.0	
Lower (<69.5)	1.0 (0.6, 1.5)	.834	0.9 (0.5, 1.5)	.636

*Note*: Unadjusted odds ratio: one determinant (bivariate logistic regression), adjusted odds ratio: multiple determinants (multivariate logistic regression).

Abbreviations: AAP, American Academy of Periodontology; CI, confidence interval; EFP, European Federation of Periodontology; OR, odds ratio.

^a^ Reference category.

For CDC/AAP severe periodontitis (Table 8), the unadjusted and significantly associated risk determinants included age ≥35 years (OR = 6.5), male sex (OR = 2.5), lower education (OR = 2.5), lower income (OR = 1.6), uncontrolled diabetes mellitus (OR = 4.6), former smoking (OR = 2.7), and current smoking (OR = 2.5); the adjusted significant risk determinants were age ≥35 years (OR = 4.5), male sex (OR = 1.9), lower education (OR = 2.0), lower income (OR = 1.7), uncontrolled diabetes mellitus (OR = 2.0), and current smoking (OR = 2.3).

**Table 8:** Unadjusted and adjusted risk associations with severe periodontitis defined using the CDC/AAP criteria

	CDC/AAP severe periodontitis, adjusted model summary: Nagelkerke *R* ^2^ = 0.325; (*p* < .001)			
	Unadjusted odds ratio		Adjusted odds ratio	
Risk determinants	OR (95% CI)	*p* value	OR (95% CI)	*p* value
**Age**				
<35 years^a^	1.0		1.0	
35+ years	6.5 (4.1, 10.6)	<.001	4.5 (2.9, 8.3)	<.001
**Sex**				
Females^a^	1.0		1.0	
Males	2.5 (1.6, 3.7)	<.001	1.9 (1.0, 3.6)	.037
**Education**				
>High school^a^	1.0		1.0	
≤High school	2.5 (1.6, 4.0)	<.001	2.0 (1.2, 3.5)	.012
**Household monthly income**				
Higher ≥5000 Saudi Riyal^a^	1.0		1.0	
Lower <5000 Saudi Riyal	1.6 (1.1, 2.4)	.024	1.7 (1.0, 3.0)	.039
**Diabetes mellitus**				
Other^a^	1.0		1.0	
No glycemic control	4.6 (2.6, 8.0)	<.001	2.0 (1.0, 3.8)	.039
**Obesity**				
Non‐obese^a^	1.0		1.0	
Obese	1.6 (0.9, 2.7)	.062	1.3 (0.7, 2.4)	.437
**Cigarette smoking**				
Never‐smokers^a^	1.0		1.0	
Former smokers	2.7 (1.1, 4.1)	.018	1.4 (0.6, 3.2)	.439
Current smokers	2.5 (1.5, 4.2)	<.001	2.3 (1.2, 4.6)	.018
**Perceived stress**				
Higher (≥42.1)^a^	1.0		1.0	
Lower (≤42)	1.5 (0.9, 2.3)	.056	1.6 (0.9, 2.7)	.057
**Perceived social support**				
Higher (≥69.5)^a^	1.0		1.0	
Lower (<69.5)	1.1 (0.7, 1.6)	.701	1.1 (0.7, 1.9)	.612

*Note*: Unadjusted odds ratio: one determinant (bivariate logistic regression), adjusted odds ratio: multiple determinants (multivariate logistic regression).

Abbreviations: AAP, American Academy of Periodontology; CI, confidence interval; EFP, European Federation of Periodontology; OR, odds ratio.

^a^ Reference category.

## 4 DISCUSSION

The current study aimed to examine the prevalence, extent, and risk associations of untreated periodontitis. The secondary objectives were to compare the periodontitis findings between younger and older individuals and test the use of a new classification system for periodontal diseases for use in epidemiological studies. To study the natural conditions of periodontitis, we selected a purposive sample of subjects who were never treated for periodontitis, either surgically or non‐surgically, in their lifetime. Compared to previous studies that focused on untreated periodontitis (Loe et al., 1978; Löe et al., 1986; Timmerman et al., 1998, 2000), our study included a broader sample as it was not limited to a specific sex group (e.g., males), a younger age group (e.g., 14–30 years) (Loe et al., 1978; Timmerman et al., 1998), or subjects from a single occupation (e.g., tea workers). We also studied several determinants for their associations with untreated periodontitis, including medical conditions, socioeconomic status, and psychosocial factors. In contrast to previous studies, our study did not use a longitudinal design, which would have allowed us to observe the onset and progression of periodontal disease. This limitation was due to the ethical concerns that would arise with studying the natural history of disease without providing available treatment (Bhopal, 2016; Jewell, 2016).

In our sample, the prevalence of AAP/EFP total periodontitis and stage III/IV periodontitis were 85.4% and 48.5%, respectively, corresponding to the prevalence of 78.4% and 31.1%, using CDC/AAP moderate‐severe periodontitis and severe periodontitis definitions, respectively. The periodontitis prevalence and extent were stratified by the sample's mean age to compare the findings in younger and older subjects. In older subjects (those ≥35 years), the prevalence of CDC/AAP moderate‐severe and severe periodontitis was 95.4% and 49.5%, respectively, higher than the corresponding prevalence in the US adult population, which was 34.3% and 7.5%, respectively (Tran et al., 2014). Periodontitis extent in older subjects in our study at two thresholds: mean % (SD) of a CAL ≥ 3 mm and a CAL ≥ 5 mm were 56.9% (30.4) and 30.9% (25.7), respectively, which were higher than the corresponding extent of 21.2% (24.4) and 6.9% (18.3), respectively, in the US adult population using the same disease thresholds (Tran et al., 2016). However, when the periodontitis prevalence and extent of our study subjects were compared to the untreated subjects in Guatemala, the findings were comparable (Dowsett et al., 2001; Dowsett, Eckert, et al., 2002; Dowsett, Kowolik, et al., 2002).

In younger subjects (those 13–34 years), the prevalence of CDC/AAP moderate‐severe and severe periodontitis was 39.1% and 13.0%, respectively, higher than those reported in other studies focused on the young general Saudi Arabian population (AlQahtani et al., 2017; Hossain et al., 2013). The periodontitis extent of a CAL ≥3 mm in younger subjects was 12.9% versus 0.9% in young military recruits from the UK (Eaton et al., 2001). Thus, the prevalence and extent of periodontitis in younger individuals in our study were substantially higher than those in other young individuals elsewhere.

Our untreated subjects had high levels of plaque, calculus, and gingivitis; similar findings were common in other untreated populations (Dowsett, Eckert, et al., 2002; Loe et al., 1978; Timmerman et al., 1998, 2000). In addition to the uniformly high levels of plaque observed in our study, several other risk determinants were significantly associated with untreated periodontitis, which highlights the fact that they have different susceptibilities to periodontitis (Loe et al., 1978; Neely et al., 2001; Van der Velden et al., 2006). The probability of periodontitis increased with age for all periodontitis outcomes regardless of the adjustments for other determinants (P. I. Eke et al., 2016; Neely et al., 2001; Susin et al., 2004). The risk of severe periodontitis was higher in males than in females with or without the adjustment for other variables, which is a confirmed association in both untreated subjects (Neely et al., 2001) and general populations (P. I. Eke et al., 2016; Susin et al., 2004). In addition, the extent of periodontitis was higher in males than in females, which was consistent among most of the age cohorts except the youngest (≤20 years), among which males and females had similar periodontitis extent. This finding of similar risk among younger patients can be explained by their similar baseline risk, which might be modified by exposures/risks later in their lives. Socioeconomic determinants, such as lower education and lower income, increased the probability of periodontitis or severe periodontitis (P. I. Eke et al., 2016; Susin et al., 2004). Uncontrolled diabetes mellitus was associated with periodontitis in the unadjusted models; however, the adjusted association was significant only with CDC/AAP severe periodontitis (P. I. Eke et al., 2016; Susin et al., 2004). This finding may indicate that the risk association with diabetes mellitus is more pronounced at a more severe periodontitis threshold as previously suggested (Albandar et al., 2018; Tsai et al., 2002). Obesity was associated with periodontitis in unadjusted models, but a significant effect was not confirmed in the multivariate models. This lack of an association between obesity and periodontitis in adjusted models could be explained by the self‐report of the condition without using an objective indicator, such as body mass index (BMI). However, this finding was similar to findings from the National Health and Nutrition Examination Survey 2009–2012 (P. I. Eke et al., 2016). Current cigarette smoking increases the probability of periodontitis and severe periodontitis (P. I. Eke et al., 2016; Susin et al., 2004). An unexpected finding was the significant association between a lower level of perceived stress and stage III/IV periodontitis. This unexpected finding might be explained by other unmeasured determinants, such as distress or inadequate coping skills (Genco et al., 1999).

Comparisons between subjective self‐assessments of oral hygiene and our objective clinical findings may indicate that our subjects lacked knowledge about their oral health, as some subjects who had a high FMPS and periodontitis extent rated their conditions as good to excellent. Study subjects reported having access to regular medical care but did not seek dental treatment, except for emergency treatment. This may highlight the need for medical doctors to convey to their patients the importance of regular dental care, especially for those with diabetes mellitus.

To study the prevalence of periodontitis, we considered the use of the new clinical definitions by the AAP/EFP in addition to the widely used CDC/AAP definitions for epidemiological studies to compare our findings with those of other studies. AAP/EFP definitions use a matrix of staging, grading, and descriptors of distribution/extent, which are mainly suggested for clinicians providing individual patient care. We tested the use of this classification in epidemiological studies in which the staging can be readily applied to estimate periodontitis prevalence and its risk associations. However, the grading is less reliable without a full‐mouth radiographic assessment, which is not routinely conducted or recommended for epidemiological studies. Previous studies either adopted staging only for population‐based data (Botelho et al., 2020) or acknowledged the challenges of using the full matrix of staging, grading, and descriptors of distribution (Germen et al., 2021; Ndjidda Bakari et al., 2021).

The implications of the current study's findings include the assessment of several risk determinants for their association with periodontitis in untreated subjects with a uniformly high plaque level. The recruitment of a wide age range of subjects allowed us to directly compare findings based on age. In addition, this study tested the application of the new AAP/EFP classification for epidemiological studies, in which detailed clinical and radiographic assessments are not routinely conducted.

Our study results have limited external validity since we targeted a specific sample of untreated subjects who were recruited from screening dental school clinics. We also relied on self‐reports of diabetes and obesity, that is, without objectively validating the measures of glycemic control or BMI.

Our suggestion for future studies is to identify subjects who never received periodontal treatment and compare their findings with those of subjects who attend regular periodontal care. We also suggest population‐based studies to estimate the proportion of untreated subjects in their sample to provide more insight into the periodontal disease burden in a population. This can be especially important providing the exceptionally high prevalence and extent of periodontitis found in this study.

## 5 CONCLUSIONS

The prevalence and extent of periodontitis were high in untreated subjects in Saudi Arabia. Risk determinants that were associated with periodontitis included age ≥35 years, male sex, lower education, lower income, uncontrolled diabetes mellitus, obesity, lower perceived stress, and current cigarette smoking of ≥10 cigarettes daily. The use of AAP/EFP staging can be applied to estimate periodontitis prevalence; however, grading may be less reliable without radiographic assessment.

## CONFLICT OF INTERESTS

The authors declare that there are no conflict of interests.

## AUTHOR CONTRIBUTIONS

*Conception, design, data acquisition, analyses, drafting, and final approval of the manuscript*: Yasmine Alawaji. *Conception, design, revising the work critically, and final approval of the drafted manuscript*: Abdulsalam Alshammari. *Conception, design, and approval of the final manuscript*: Nesrine Mostafa. *Revision of the workflow critically at all stages and approval of the final manuscript*: Ricardo Carvalho. *Critical contribution to the design, analyses, and approval of the final manuscript*: Jolanta Aleksejuniene.

## Data Availability

The data subject to restrictions by King Abdullah International Medical Research Center in Riyadh, Saudi Arabia.
